# Perceptions on using surplus embryos for the treatment of Parkinson’s disease among the Swedish population: a qualitative study

**DOI:** 10.1186/s12910-022-00759-y

**Published:** 2022-03-04

**Authors:** Åsa Grauman, Jennifer Drevin

**Affiliations:** Centre for Research Ethics and Bioethics, Uppsala University, Box 564, 751 22 Uppsala, Sweden

**Keywords:** Surplus embryos, Public perceptions, Parkinson’s disease, Drug development, Moral concerns

## Abstract

**Background:**

Human embryonic stem cells are currently used for developing treatment against Parkinson’s disease (PD). However, the use of ES cells is surrounded with moral concerns. Research regarding the public's attitudes can form an important basis for policymaking. The aim was to explore the perceptions of the public on using donated human embryos for developing treatment of Parkinson’s Disease.

**Methods:**

Semi-structured individual qualitative interviews were conducted with 11 members of the general population in Sweden. Interviews were analyzed with thematic content analyses.

**Results:**

Four categories and additional sub-categories; Different views on the embryo requires delicacy, Using embryos to treat Parkinson’s disease, Doing things in the right way, and Communication, media, and public opinion. In general, respondents were positive towards the usage of embryotic stem cells to treat PD, but the usage were conditioned and specific terms were demanded. Informed consent from both donors were required and delicacy and sensitivity when working with embryos were needed.

**Conclusions:**

It was perceived better to use surplus embryos to treat PD increase is than to discard them, also among those who perceived the embryo as “a potential life.” The participants raised several conditions under usage for treatment should be allowed. Even if the embryos otherwise are going to be discarded, usage requires informed consent from the donating couples.

## Introduction

Parkinson’s disease is a chronic progressive disorder, where the nerve cells that produce dopamine are destroyed, which reduces motor control. The disease can seriously impact the possibility of performing daily activities, and it decreases an individual’s independence. Currently, there is no cure for Parkinson’s disease; instead, treatment aims to reduce symptoms and maintain a quality of life. Development of effective treatment therefore is crucial to help patients and their families.

Embryonic stem cells (ES cells) are immature, pluripotent cells that have the ability to develop into any specialized cell and divide an infinite number of times [1]. Generation of ES cells involves extracting cells from the embryo on the fifth day after conception, which often causes the embryo to be destroyed. The embryos used to generate ES cells are those that have been left over and donated by couples who have undergone fertility treatment to achieve a pregnancy. In parallel with the development of ES cells, it is now possible to produce induced pluripotent stem cells (iPS cells) by reprogramming specialized cells [2, 3], such as skin cells. By controlling the differentiation of ES cells and iPS cells, researchers hope that both can be used in medical treatment to replace the cells that have been damaged or have died. Parkinson’s disease is one of the conditions, where the development of treatment has come the furthest.

Unlike iPS cells, the use of ES cells is surrounded by certain moral concerns because embryos are destroyed during production, and because the manufacture of drugs as well as medical treatment involve commercialization. Some concerns have previously been expressed that the sale of ES cell therapy risks leading to embryos and ES cells being considered as commodities, with a lack of respect for human life [4]. The view of the embryo’s moral status varies, depending on the outlook on life and has previously been the subject of extensive discussions [5–7]. By using iPS cells, one could avoid the moral concerns that surround the use of ES cells. However, research on iPS cells and ES cells is ongoing in parallel, and some researchers believe that treatment with ES cells may be available for medical treatment of patients at an earlier stage than iPS cells.

When treatment with ES cells will be ready to be introduced on the market, it is reasonable to assume that it will lead to discussions on whether commercialization of these cells should be allowed for the treatment of patients, or under what conditions they should be allowed. In a previous cross-sectional study of the attitudes toward aspects of embryo donation in Sweden, about half of the participants were positive about donating for research purposes [8]. However, the purpose of donation differed from this study. The disease area was not specified, and no deeper reasoning was captured by the study, which makes it difficult to understand the arguments and motivations behind their attitudes. This study aims to explore the perceptions of the public on using donated human embryos in treatment for Parkinson’s Disease.

## Methods

### Design

The study was designed as a qualitative, semi-structured interview study.

### Research ethics

The study was approved from the Swedish Ethical Review Authority for conducting human interviews, before the data collection started (Dnr 2019-06539). All participants provided written informed consent to participate in the study, including for collecting background variables, and being interviewed and recorded. Data were presented in such way that no individual can be identified. All the methods were carried out in accordance with relevant national and international guidelines and regulations.

### Context and settings

Currently, in Sweden, couples undergoing in vitro fertilization (IVF) can choose either to discard their embryos or to donate the embryos for research purposes or to donate to other couples for reproductive purposes. If the embryos are not donated after the maximum storage time of ten years has elapsed, the remaining embryos will be discarded.

This study was part of a research project with the overall aim to investigate how the public, couples with cryopreserved embryos and patients with Parkinson's disease view the use of donated embryos for medical treatment, using both qualitative and quantitative methods.

### Participant selection

Members of the public were recruited through choirs in one of the larger county councils of Sweden. Recruitment via choirs were chosen for pragmatic reasons to reach individuals of various sexes, ages, and religious beliefs, since choirs are popular in Sweden and attract citizens of various sex and ages. Approximately 44 choirs with adult members were contacted via e-mail and/or phone, whereof 19 choirs were reached. Choir leaders or contact persons were contacted and asked to distribute information to their choir members. Ten choirs agreed and nine declined. The ten choirs had different profiles, which included individuals of various sexes and ages, e.g., student choirs, church choirs, and more general choirs. The 10 choirs had 315 members in total. Members interested in participating were asked to contact the researchers. We were contacted by 11 persons that were interviewed. The background characteristics of the participants are shown in Table 1. The majority of the participants were women, highly educated, and were working or retired. The mean age was 57 years.

**Table 1:** Participants’ (n = 11) characteristics, presented using frequencies (*n*) and percentages (%) or mean (M) and standard deviation (SD). It was possible for participants to choose more than one occupation

	*n*/M	%/SD
Gender		
Female	9	82
Male	2	18
Age (years)	57.4 (range 21–72)	15.8
Country of birth		
Sweden	8	73
Other	3	27
Occupation		
Working	5	45
Student	1	9
Sick leave	1	9
Retired	6	55
Completed level of education		
Upper secondary school	2	18
College/University	9	82
Use of medication		
Daily	7	64
1–3 days a month	1	9
Less than one day a month	3	27
Medical training^a^		
Yes (Physician, nurse, Biomedical analytic	5	45
No or did not say	6	55
Experience of Parkinson’s disease^b^		
None	6	55
Have family members, relatives, or friends with PD	4	36
Have worked with PD patients	1	9
Conception of life^b^		
Christian	1	9
Raised in a Christian tradition but not religious, nor atheist	6	55
Humanist	1	9
Agnostic	1	9
Atheist	2	18

^a^Brought up spontaneously during interviews

^b^Was asked during interview

### Data collection

The participants were asked to respond to a web questionnaire concerning their background characteristics (Table 1). Secondly, they were asked to read some information about human embryonic stem cells before the interview, which introduced the participants to the topic.

The interviews were held by phone during August 2020–March 2021. The interview guide is presented in Table 2. All interviews were performed by JD, a registered nurse with previous experience in qualitative research. The interviews lasted between 32 and 81 min, with a median of 55 min. All participants received a movie ticket as an incentive for their participation. Data were considered saturated after the eleventh interview. No new themes emerged and no new information was recurring.

**Table 2:** Interview guide used in interviews

Warm-up question	Do you have, or have you had, anyone close to you with Parkinson’s disease?
Warm-up question	Have you previously heard of medical treatments using cells taken from embryos?
	If you think of when you first heard of medical treatments using cells from embryos, what were your first spontaneous thoughts?
	What is your view on using embryos for treatment of Parkinson’s disease?
	What is your view on that donated embryos are destroyed in the process?
	What is your view on that companies may profit from selling these medical products?
Follow-up question	In this question, what is important to you? Interests/values? To whom?
Follow-up question	What affects your outlook on this matter, do you think? Any beliefs?
	If you reflect, on what you have told me so far, what aspects are most important to you concerning using surplus embryos to treat Parkinson’s Disease?
	Have you changed your view on this matter during the interview?
Summarize the interview	[Interviewer sums up what the participant has described so far]
	Have I misinterpreted something, or do you want to add something?
	The purpose of this interview was to explore your views on using donated embryos for medical treatment and any values or interests related to it. Is there something you think of that has not been brought up yet?

### Analyses

The inductive analysis was performed using thematic content analysis [9] and performed by both the researchers (JD and ÅG). After reading the transcribed interviews, open coding was applied, which entails making a summary statement (a code) of what has been said. The first interview was coded by both researchers independently. The coding was then compared, and deviations were discussed. The remaining transcripts were then divided between the two researchers. All the codes were then listed, and duplicates crossed out. Similar and overlapping codes were then grouped together into categories. During the analyses process, the researchers discussed the categorization and went back to the text for a constant search for meaning of the data. The software Atlas.ti 9 was used to organize the codes and categories.

## Results

The analysis resulted in four categories and additional sub-categories (Table 3). Overall, the respondents were positive toward using embryos for drug development, but the terms of use were conditioned, and the sensitivity of the matter recognized. Different views could be expressed from the same individual by going back and forth between the different perspectives.

**Table 3:** Categories and sub-categories (below each category)

Categories	Different views on the embryo requires delicacy	Using embryos to treat Parkinson’s disease	Doing things in the right way	Communication, media, and public opinion
Sub-categories	Perceptions of the embryo and its value	Usage is beneficial and increases utility	Couples permit and informed decisions	Importance of transparency and communication
	Awareness and respect for moral sensitivity	Purpose of origin and prioritization of usage	Following rules and ethics and treating the embryo with respect	Negative debate may harm donors and research
		If all else equal, choose IPS	Profit and economic compensation to the donors	Listen to the opinion when making policy
			Concerns about future use of embryos	

### Different views on the embryo requires sensitivity

#### Perceptions of the embryo and its value

The participants had varying views on the embryo, which was also reflected in their choice of words when describing the embryo, e.g., a cell lump, living cells, a potential life, or a child. Some did not perceive the embryo as a life and did not think it was covered by human rights, since it does not have consciousness and could develop into anything at that moment.

P10: Yes, I see it as, well really, as a cell clod or what to call it. But I don’t see it as a life.

P11: There is no consciousness; there is no own personality or drive or intention of any kind. Thus, it is a biological process that has not become conscious.

Other participants thought that everybody has the right to life, including embryos; furthermore, they thought that it was a potential life that should be treated with respect. They expressed a feeling of unease associated with the thought of embryos being disposed. Some of the participants mentioned the special sentimental value that the embryo has had for the couples, who intended for it to become a baby, and who invested a lot of feelings and hopes into it.

P4. That everyone should have the right to a life if one has come to be, sort of. That everyone has the right to a life no matter how small you, sort of, are. If you are an embryo, it is the beginning of a life; the beginning of a human being. What would happen to that human if it had been allowed to live? […] it is then that you, it is now like this, kill a life.

The age of the embryo and how far it had developed was recognized as important, and some made a clear distinction between the embryo and a fetus. However, most were uncertain regarding exactly when life begins, and one participant referred to it as being an empirical question. Others talked about the embryos in terms of being a new raw material and a resource for healthcare and that it has value based on that.

#### Awareness and respect for moral sensitivity

Regardless of how the participants themselves felt about the embryo and what they perceived it to be, most acknowledged the fact that handling embryos was a sensitive matter, due to the different values held by people. Researchers and members of the industry should take note of this in order to show sensitivity and respect.

P11: I can see that there are concerns grounded in your values and life views. This is in sharp contrast to how I think and how many others in a secularized society think, but it is an ethical conflict that those who engage in this kind of research still must be aware of and take a position on, as it has consequences also for the persons who choose to donate an embryo.

### Usage of embryos to treat Parkinson’s disease

#### Beneficence & increased utility

Overall, the participants were positive about using ES cells for the development of treatments for patients with PD. It was perceived as the right thing to do, to help humanity, and to improve the patients’ lives. They acknowledged the need to find effective treatments for these patients. Some participants had witnessed hopelessness and suffering, having had family members or friends with PD, or from working within healthcare. Many of the participants expressed an interest in research and a trust in the scientific community, which they thought influenced their attitudes. Several participants thought their attitudes were influenced by their life experience.

P4: If you then can, with the help of embryos and its cells, really create an opportunity, in this case Parkinson's, to perhaps cure or find ways to make life a little easier, then it is worth its weight in gold.

Using the embryos for drug development was perceived by some as giving meaning to the embryos’ life, and what “the embryos would have wanted.” For some, the use of embryos was completely unproblematic since they did not perceive them as a living thing and because the couples no longer needed them. Some pointed out that the rejection of embryos is a natural process, which happens in the body all the time. Some were positive about donating embryos themselves. The participants hoped that ES cells could be helpful also for patients with other diseases.

P1: What I think about using human cells, I have no problem with that at all really. […] It was not associated with any question of conscience whatsoever.

P3: It must be like, ethically, more correct, or that is in any case for me, ethically correct, that they can sort of contribute to something in the research. It creates a meaning for these children's lives, you can imagine. That’s how I make sense out of it […] that it must be, sort of, ethically better to give a task to these embryos, than to throw them in the trash, which is the alternative otherwise.

#### Priorities related to the purpose of embryo creation

Many participants thought that the use of embryos is justified because the surplus embryos would otherwise be discarded. Several participants mentioned a priority scheme about what to do with surplus embryos from IVF, in some cases based on the purpose of the embryo creation. Hence, it is better to donate the embryos to other couples before using them in drug development. Secondly, they should be used for drug development and lastly, they should be disposed. However, one participant thought that since these embryos are created for the purpose of giving the donors a baby, it is most reasonable to dispose of the extra embryos when that purpose is fulfilled, instead of using them. The participant advocated that it would be better to create embryos for the purpose of drug use, since they would, then, never have been intended to become babies. Other participants valued helping couples to have a baby more than drug development and disposal, regardless of the purpose of the embryos.

P2. The embryo is surplus and that you would throw it away, anyway. The only thing I can think of that is more important to do with the embryo is to become a baby, but I assume that not all embryos they have can become babies, and then it's just great that you can do research with it. […] I guess I probably would, if I have an embryo over… that I would rather give it to the couple who want a child, than to this that goes to research, but I guess there are so many embryos that it is enough for both.

P8: “For these leftover embryos… if you cannot use them to create a new child for someone who cannot, if it would be an alternative, then I would prefer it because I would think that is more important. But, I really have no ethical basis for that, I would say, not directly in any way. The drug in the end, I have no problem with, but my reasoning is, if I have any opinion at all, it is about which embryos you use to make these cells. It is important for me that they are donated and not primarily can be used to create a new child or something like that.”

#### If all else is equal, use IPS

It was perceived as acceptable to use both iPS-cells and hESCs for the development of a treatment for PD. If choosing between them, the decision should be made based on the efficiency and side effects of the treatments. If equal, iPS was preferred over hESCs since it was perceived as more practical and less of an ethical issue compared to using hESCs. The risk of negative reactions in society was expected to be lower when using IPS cells. Furthermore, the burdensome process of collecting and fertilizing eggs could be avoided. The decision to donate would also become easier, since it only involves one person. Some thought that hESCs are used today due to the lack of other options, but the day when other solutions are available, embryos will not be used anymore.

P3: It is my opinion, and it would make much more sense for me ethically, if there were equivalent alternatives. Then, I think you should thank these embryos for what they have done [with them], but now, one does not need to use them anymore. […] I think it is fundamentally good but uncomfortable, so I think it would be much better if one does not use them later.

P9: If they have the same effect and are just as efficient to produce and that whole thing, then it is clear that you might think that, well, use skin cells, instead. Because then, you get away from this little moral or ethical conflict that many have and that it may be more okay, research in general for everyone. […] So, if you can only use skin cells and it has the same effect, then I think the research would benefit from it by avoiding that contradiction or the conflict with those who think it is unethical. For my part, I still stand by the fact that I think both cells are okay to use.

### Doing things in the right way

#### Couples’ permission and informed decisions

The participants stressed that the most important thing when it comes to use of embryos was that the couples have given their permission. The decision to donate their embryos must be informed and free from pressure and influence of others. Information to presumptive donors should be objective and neutral. The couples should have enough time to contemplate their decision based on what is right for them. Since people’s beliefs and conception of life would influence how they view the matter, the decision to donate is up to every single person.

Some participants made parables with the regulations of organ donation in Sweden (an opt-in procedure), with the exception that there are two people involved in the decision, where both need to approve the donation. It was perceived as positive for the couple to have the option to choose what their embryos should be donated to, to childless couples or to research, and the possibility of choosing the research area. Some perceived it as if the parents decided for the embryo that could not make his/her voice heard in this situation.

The participants also brought up patients’ possibility to choose treatment and to decline treatment developed using ES cells. One participant thought that although it is the couples’ free choice to donate, they have a duty to do so since they have received help from the healthcare. One participant found it sufficient for couples to actively opt-out if they do not wish to donate the embryos, instead of giving their permission.

P6: You actually have to accept that people have to get enough information to be able to make different decisions, and you have to accept if people make decisions that you do not like yourself, which can be a little difficult to digest, but there is a right for people to decide for themselves.

P8: And I am principally not against it as long as it's based on voluntary donations.

#### Following rules, regulations, and ethics

The participants emphasized the importance of “doing things right,” according to rules and regulations, and to act ethically. Some thought it was important that the embryos were handled with respect, even if it was not easy to define what exactly respectful handling involves. The interests and safety of the donors should be carefully protected, such as their health and genetic data. The treatments developed from embryonic stem cells must be properly tested and the side effects studied, just like all other drugs, before being put on the market. Several expressed high trust in experts and researchers, feeling that they strive to do good. Many brought up the importance of making future products available. The pharma industry had a responsibility to make their drugs accessible for everyone in need of it, and not only for rich people and countries.

P6: I think that there are many requirements to create a donation process that takes into account the donor's interests and safety, and the quality and safety of the donated material.

P7: Mmm, for me, it is like so, that they were handled disrespectfully, it would not feel okay. I think about performing lots of unnecessary tests on them...

One participant, a physician herself, expressed empathy with the couples that sought medical care to receive help with achieving a pregnancy. She raised considerations if the information and decisions about embryo donation could harm them by inflicting “ethical burden,” from which they should be shielded. Likewise, it was suggested that future patients should only be informed about efficiency and side effects and not that the drug is developed from ES cells. Patients should not be bothered with ethical aspects of a drug since it could inflict harm.

P3: I think that there are patients and relatives who would also have the same discomfort or how to put it, that this comes from a fertilized egg that has been destroyed, which is then a potential life, […], that this is an ethical burden to be thinking about this and having to relate to the issue of human life and things like that […]. This combined thought burden is something that sick patients in particular could be allowed to avoid, the overall ethical thought burden of this if it is right with IVF, if one like, if it was right to get rid of the child, and if it was right to destroy the cell and so on.

#### Profit and economic compensation to donors

The profit that companies stand to make from drug development from embryonic stem cells was perceived, by some, as secondary to helping patients, and something that was inevitable for drugs to be developed, and by some not different from any other drugs on the market. Others expressed that there is a moral limit to how much profit one can make from a person’s suffering and had to do with whether a lucrative market increases the risk of donors being exploited. It is reasonable that companies that make huge profits on the suffering of humanity give something back to society. To make things fairer, some thought that the donors should be compensated for their efforts. However, economic compensation may risk further exploitation of vulnerable groups and cause donors to make decisions based on economic gain, instead of their values. Some participants therefore thought that donation should only be done based on altruism. Others thought that a small compensation, comparable to blood donation, would be reasonable.

P2. I cannot say that I am against this with embryos and research because maybe uhm some pharmaceutical companies get rich of it […]: I cannot influence that, and it is still more important that those who have Parkinson's, that they are helped.

#### Concerns about future use of embryos

Although the participants were accepting of the current conditions regarding donation of surplus embryos from IVF, some brought up concerns concerning future use. Most participants were negative toward large-scale trade with embryos and creating “embryo factories.” However, some did not think it was acceptable to create embryos only for the purpose of drug development, since they saw it as “playing with life.” Furthermore, intentionally collecting more embryos than needed as part of the IVF situation only for the purposes of research was not deemed ethical. There were also concerns about situations where the embryos got into the wrong hands, where unnecessary testing would take place, or where the embryo would be developed into a fetus or used to design babies. Nonetheless, many thought that these concerns were unrealistic, at least in Sweden.

P8: But when it comes to exploitation, then I still imagine, like, so that one does not become an embryo factory where embryos are sort of, like, harvested from women like in an assembly line, if you understand what I mean.

P1: No, but if it ends up in the wrong hands and the embryo grows and becomes a fetus and yes, that it ends up in the wrong hands and that you are performing research on small fetuses.

### Media, communication, and public opinion

Importance of transparency and communication of the research results.

The participants thought it was important for researchers to be transparent and communicate news about the progress of the research. Communication can increase knowledge and awareness of the possibilities involved in stem cell research. Many participants thought that talking more about ES cell research would increase permissiveness and openness about the ongoing research.

P5: To inform and educate the public, so that they understand that this is a good idea. Ehh, because it is, in other words, stem cells, so they are very important to have because they are needed to do a number of things.

#### Negative debate may harm donors and research

There were concerns about polarization due to an unbalanced reporting in media. The participants wanted to avoid a judgmental pro-life debate since it could harm the donating couples and induce guilt and “ethical burden” on their shoulders. A negative debate may also inhibit valuable research. The participants talked about the importance of accepting and respecting different views held by members in society in the public debate.

P3: I think that there is another ethical aspect if you were to discuss this a lot in the media and that is namely, how it is, how it will be, as I think, I have personal experience, these ailments in other words, if the parents have to, so to speak take a stand, or then, to not, well to not, get rid of, their fertilized embryos. Then, I think it would be both at a socially beneficial level, where there could be a suspended stem cell research and that for the parents who have great benefit from IVF; they would then end up in a terrible situation. So there, I think to have a reasonable discussion in public.

#### Listen to the opinion when making policy

It was perceived as important to listen to and inquire about the public opinion before making policy decisions on using embryonic stem cells. Current norms should guide decisions. One participant acknowledged that today’s society has already accepted that IVF means an abundance and disposal of surplus embryos.

P9: Regardless of the fact that I think you should do the research, it is important that you have society or the majority of the population with you.

## Discussion

The participants were, overall, positive toward using ES cells for developing a treatment for patients with PD. However, the usage was problematized and conditioned. The participants showed moral values in line with the four biomedical ethical principles; beneficence, non-maleficence, autonomy, and justice [10]. Maintaining the autonomy of the donors was strongly emphasized by the participants. Even if the participants themselves perceived the usage of embryos as unproblematic, they thought that informed consent was required from both donors. Several of the participants also pointed out that the possibility of choosing a specific purpose for the donation was preferable.

Some found embryo donation to be completely unproblematic, while others had ethical concerns connected to their perception of the embryo, in terms of being a “potential life.” These perceptions of the embryo are very similar to findings in similar studies [11]. Using the embryos was perceived as being beneficial and better than discarding them. However, many participants valued the opportunity to donate to infertile couples as opposed to donating to research, which has been reported repeatedly before, both among the public and among patients [8, 12, 13]. The participants who viewed the embryo as a potential life were still positive about donating, since they considered the benefit of helping PD patients as more important than the negative aspects related to using the embryos. The specific context of PD was considered when balancing these benefits and harms, in terms of suffering and hopelessness of not having an available cure. Some thought that ES cells will no longer be used when other treatments are made available to these patients. Previous studies have not been disease-specific [8, 14]; therefore, they may have misjudged the people’s attitudes about donation to a specific research area, since it is possible that attitudes differ between disease areas due to severity and available treatment. Being specific about disease areas and the current situation for the patient group, therefore, is important, both in attitude research and in the informed consent procedure when approaching potential donors in a clinical setting.

The importance of not inflicting harm on embryos, donors, patients, and to the research itself was also raised. Although the embryos can be used for research purposes, they must be handled with respect, e.g., not performing unnecessary testing. The interest and security of the donors should be protected and exploration avoided. Information and an eventual negative public debate about using or discarding the embryos could harm donors and future patients, by inflicting “ethical burden” on them, and obscure valuable research. However, in year 2001 there was an ongoing public debate in Sweden about ethical aspects about the use of surplus embryos. The following year, 92% of couples who underwent infertility preferred donating their surplus embryos for stem cell research rather than letting them be discarded, which was a small increase compared with the year before [15]. Likewise, the health professionals working in the clinic thought it was easier to inform couples about donating the embryos. This may indicate that having discussions in society can lead to more awareness and openness toward donation of embryos for research [15].

In the discussions on commercialization and the role of the pharmaceutical industry, a justice perspective was raised. It was viewed as important that the treatments developed must be available to all patients in need, and not only the privileged ones. Since the industry profits from the embryos donated by the couples, it is fair that they give something back to society. Similarly, some thought it was unjust that the industry makes a profit, while the donors are left without compensation for their burdensome effort. However, compensation to donors was problematized since it may increase the risk of exploitation by the donors.

### Strengths and limitations

In qualitative research, the aim is to gather a variety of perceptions, which is why you often strive to recruit a heterogeneous sample. However, it is heterogeneity in views that is sought rather than heterogeneity in people [16]. Therefore, it is important to consider how people are expected to differ in their views. To recruit participants for the study, we reached out to choirs, as a pragmatic way of reaching individuals of various sexes, ages, and religious beliefs. Although choirs are very popular in Sweden and can have different profiles, it is likely that some members of the public cannot be reached via choirs.

Men were underrepresented in our study; however, men have, in general, been more positive about allowing embryo donation for research [8, 14], which indicates that the results may not have differed a lot by including more men, since the participants overall were positive. Health staff have also been more positive than the public in previous studies on donating to research [14]. No systematic assessments were carried out on participants’ occupation or medical training, but the participants spontaneously mentioned it during the interviews. Being scientifically oriented and having trust in experts were also something that the participants themselves mentioned as explanations for their positive attitudes about donating to research. Feelings of reciprocity toward science and medicine and having high levels of trust in the medical system have also been reported among IVF patients who donated embryos for research [13]. Previous studies have also found that one’s country of birth [17], education [18], and religious beliefs [19] influence the likelihood of donating to research. In our study, most participants were Swedish born, highly educated, and did not express strong religious beliefs. Including people that differ in these aspects may have resulted in further variations in attitudes and perceptions, which is a limitation of this study. Nonetheless, the participants did differ in sex, age, perception of the embryo, medical training, and in their experience, in terms of having family members or friends with PD. The results from a qualitative study cannot be generalizable, but with a thorough description of the participants, it is possible to achieve transferability.

## Conclusion

Using surplus embryos to find a treatment for PD was perceived as better than discarding them, also among those who perceived the embryo as “a potential life.” The participants had several conditions for being able to use embryos for development of a treatment. Even if the embryos otherwise are going to be discarded, usage requires informed consent from the donating couples.

## Data Availability

The datasets generated and/or analyzed during the current study are not publicly available since it contains sensitive information, but are available from the corresponding author on reasonable request.

## Abbreviations

ES cells: Embryonic stem cells
iPs cells: Induced pluripotent stem cells
PD: Parkinson’s disease
IVF: In vitro fertilization
